# Three-port single-intercostal versus multiple-intercostal thoracoscopic lobectomy for the treatment of lung cancer: a propensity-matched analysis

**DOI:** 10.1186/s12885-018-5256-y

**Published:** 2019-01-05

**Authors:** Zixiang Wu, Qi Wang, Cong Wu, Tianwei Zhan, Lingjun Dong, Shuai Fang, Xuyang Peng, Lian Wang, Saibo Pan, Ming Wu

**Affiliations:** 1 Department of Thoracic Surgery, The Second Affiliated Hospital, Zhejiang University School of Medicine, No. 88 Jiefang road, Hangzhou, Zhejiang Province China; 2 Department of Medical Quality Management, The Women’s Hospital, Zhejiang University School of Medicine, Hangzhou, China

**Keywords:** Three-port single-intercostal, Video-assisted thoracoscopic surgery, Lobectomy, Lung cancer

## Abstract

**Background:**

In this retrospective study, we aimed to demonstrated that three-port single-intercostal (SIC) thoracoscopic lobectomy is an effective choice for lung cancer by comparing the perioperative outcomes of patients with non-small-cell lung cancer treated with three-port SIC and conventional multiple-intercostal (MIC) thoracoscopic lobectomy.

**Methods:**

From January 2013 to January 2018, 642 non-small-cell lung cancer patients underwent thoracoscopic lobectomy via a three-port SIC or MIC technique. Propensity-matched analysis incorporating preoperative clinical variables was used to compare the perioperative outcomes between the two groups.

**Results:**

The first 20 patients were excluded to account for the learning curve effect in the SIC group. Propensity matching yielded 186 patients in each group. A small percentage of patients had major morbidity, including 4.8% in the SIC group and 6.5% in the MIC group; there was no significant difference between the two groups. Although the total number of lymph nodes harvested (25.3 vs. 23.8, *p* = 0.160) and stations removed (6.5 vs. 6.7, *p* = 0.368) were similar between the two groups, more subcarinal lymph nodes were removed (6.9 vs. 5.2, *p* < 0.001) in the SIC group than in the MIC group. Furthermore, other perioperative outcomes in the SIC group were not fewer than those in the MIC group.

**Conclusions:**

Both techniques are acceptable for the treatment of non-small-cell lung cancer. Three-port SIC VATS lobectomy can provide an alternative procedure in thoracoscopic surgery.

## Background

Over the past two decades, lobectomy via video-assisted thoracoscopic surgery (VATS) has been accepted as an effective and safe treatment for patients who are diagnosed with non-small-cell lung cancer (NSCLC) [1, 2]. Compared with conventional thoracotomy, thoracoscopic lobectomy is considered to produce fewer complications, require shorter hospital stays and cause less pain for patients [3, 4]. However, the techniques used in the procedure vary among surgeons, and there is no standard approach [5, 6].

Conventional three-port multiple-intercostal (MIC) lobectomy has been applied by most surgeons for thoracoscopic lobectomy. Employing standardized three-port MIC lobectomy can allow different angles for lymphadenectomy and bronchovascular dissection [7]. Disadvantages of this approach include possible injury to multiple intercostal nerves and creation of a torsion angle in the target tissues for the observation port relatively low in the intercostal space [8, 9]. In recent years, a modified technique, three-port single-intercostal (SIC) thoracoscopic lobectomy for NSCLC [10, 11], has been adopted in our department. This technique can reduce the usage of the intercostal space, and different angles for lymphadenectomy and bronchovascular dissection are made possible. However, three-port SIC lobectomy and conventional three-port MIC VATS lobectomy have never been compared. We hypothesized that patients with NSCLC treated with SIC VATS lobectomy would have comparable perioperative outcomes to patients treated with MIC VATS lobectomy. This study presents our experience performing SIC VATS lobectomy based on a propensity-matched analysis.

## Methods

We reviewed patients (January 2013–January 2018; *n* = 642) treated with three-port SIC and MIC thoracoscopic lobectomy in the Department of Thoracic Surgery of the Second Affiliated Hospital of Zhejiang University. This study was reviewed and approved by the Ethics Committee of the Second Affiliated Hospital of Zhejiang University, and written informed consent was obtained before surgery from all patients. The inclusion criteria for VATS lobectomy were a clinical stage of T1-T3, N0-N1, and M0 without a previous history of malignancies. To minimize surgeon bias, all surgeries included in this study were performed by two senior consultant surgeons who were the first to perform VATS lobectomy in our department in the same year (2003). Moreover, these two surgeons had completed at least 2 h minimally invasive surgeries per year during the previous 15 years, and the recorded surgeries in the last 5 years were reviewed.

The first three-port MIC VATS lobectomy was performed in our department in 2003. With increasing experience, one surgeon began to employ the SIC technique instead of the MIC technique in November 2014, whereas the other surgeon still performed three-port MIC due to individual preference. The first 20 cases were excluded to account for the learning curve effect.

All the patients underwent a preoperative examination, which included respiratory function tests, computed tomography scanning of the chest, brain magnetic resonance imaging and cardiologic assessment. Clinical and demographic data, including age, sex, smoking history, forced expiratory volume in the first second (FEV1), tumour diameter and location, and pathologic stage, were recorded. The operative time, estimated volume of blood loss, length of hospital stay, chest tube removal, complications and mortality within 30 days were collected. Pathologic stage was determined based on the American Joint Committee on Cancer staging system, 8th edition.

### Surgical technique

All patients were in the lateral decubitus position with the diseased side facing upward, and all surgeries were performed under one-lung ventilation. The surgeon and assistant stood on the ventral and dorsal sides of the patient. A 10-mm, 30° thoracoscopic instrument and a straight endoscopic instrument were required during the surgery. A soft plastic wound protector was routinely applied without rib spreading.

In the conventional MIC thoracoscopic lobectomy, a 1 cm incision was made at the midaxillary line in the eighth intercostal space as the observation port. A 2 cm to 3 cm mini-thoracotomy was made at the fourth or fifth intercostal space along the anterior axillary line, and another 1 cm incision was created at the tip of the scapula. After confirmation of the target lobe, the pulmonary vein, artery, and bronchus were divided and sectioned with endoscopic staplers.

**Fig. 1:**
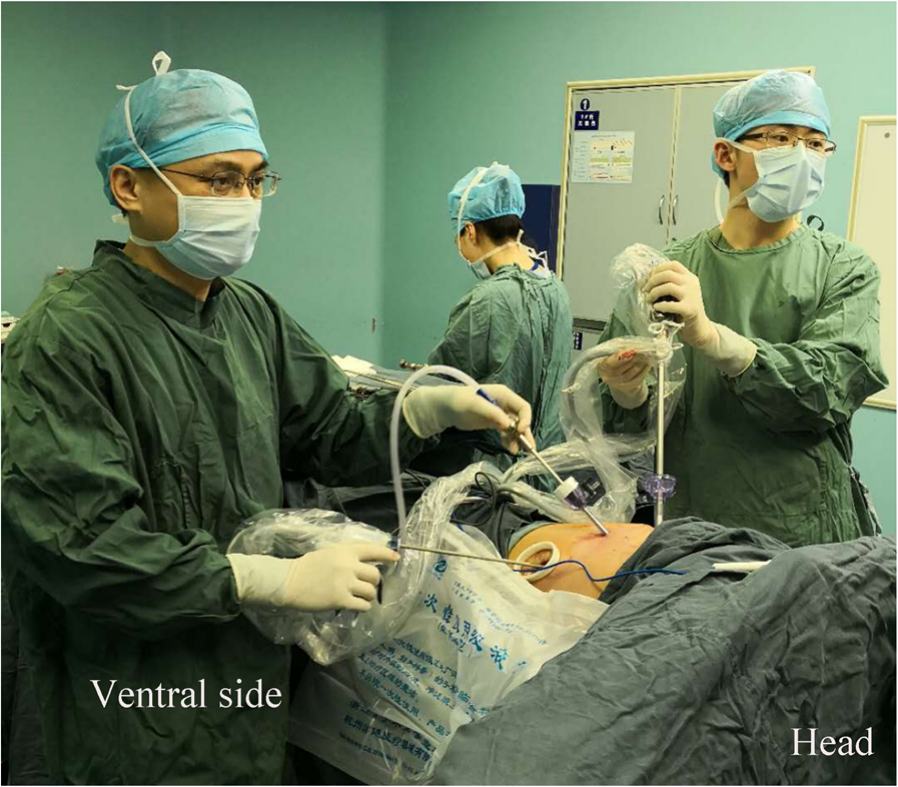
Three-port single-intercoastal thoracoscopic lobectomy for lung cancer

We have previously described the three-port SIC surgical technique [10, 11]. In brief, three operative ports were made at the single sixth or seventh intercostal space as follows: a utility port (2 cm - 3 cm in diameter) for endo-instruments was made at the anterior axillary line, a secondary work port (0.5 cm) was made at the midaxillary line for the biopsy forceps or the grasper, and a camera port (1 cm) was made at the posterior axillary line. The assistant stood at a higher position than the senior surgeon to make it easy for the assistant to maintain the camera in a vertical plane and provide direct visualization (Fig. 1). The surgical steps in the SIC technique are similar to those in MIC thoracoscopic lobectomy because the operating angle is similar, and no new instruments are needed.

**Fig. 2:**
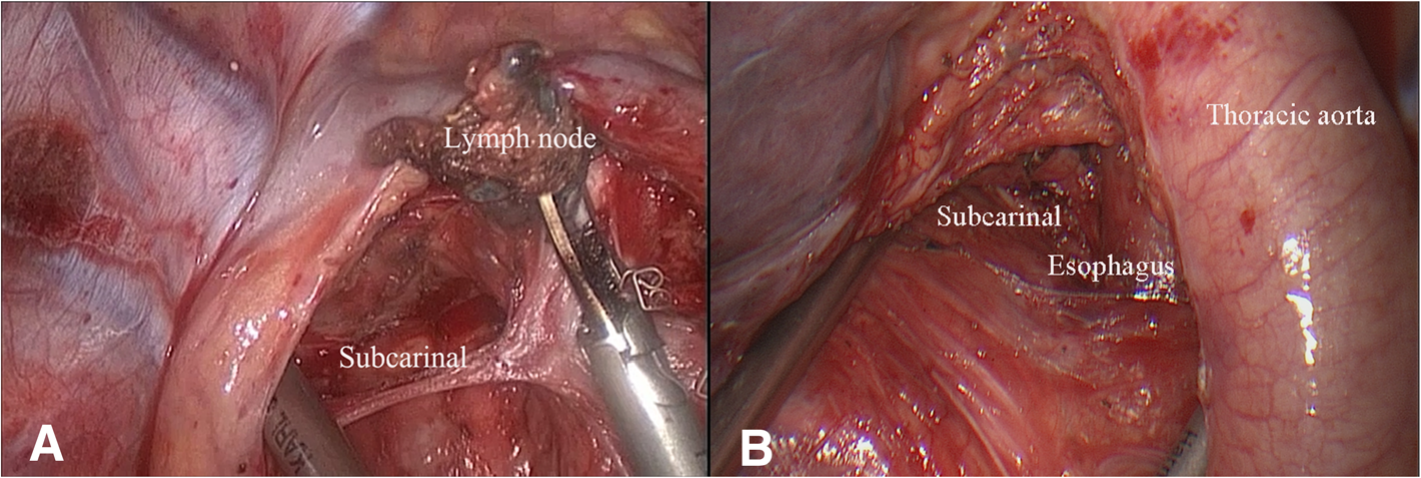
**a** Visualization of the right subcarinal lymph node dissection during three-port single-intercostal thoracoscopic lobectomy; **b** Visualization of the left subcarinal lymph node dissection during three-port single-intercostal thoracoscopic lobectomy

Systemic mediastinal lymphadenectomy, including the subcarinal lymph node and at least three lymph stations, was routinely performed in all surgeries (Fig. 2). Before September 2016, the main chest tube (26-Fr) was inserted through the utility port and connected to an underwater sealed bottle for postoperative drainage; the criteria for chest tube removal were no air leakage and a drainage volume of less than 100 mL per day. After that time, a common stomach tube (14-Fr; Terumo Medical Products Co., Ltd., China) connected to a plastic bag was added as an assistant chest tube through the secondary work port. The main chest tube (26-Fr) was removed when no air leakage was found. The assistant chest tube was then removed when the drainage volume was less than 200 mL per day. Patient-controlled intravenous analgesia was used for postoperative analgesia before 2014. Subsequently, a patient-controlled paravertebral block [10] for continuous regional anaesthesia combined with administration of an oral pain medication (ibuprofen, 400 mg) twice a day was used for patients who underwent SIC VATS lobectomy. Complications in the hospitalization period were all treated with appropriate medication. Patients were discharged only if all the chest tubes were removed and after assessment of the patients’ well-being by senior doctors.

### Statistical analysis

Clinical information of all selected patients was gathered from a highly maintained database by the authors. We employed a one-to-one propensity score matching analysis for the comparison of three-port SIC and MIC thoracoscopic lobectomy because the treatment assignment was not random. The dependent variables in the logistic regression model were age, sex, forced expiratory volume in 1 second (FEV1), tumour diameter and location. Patients were then matched by using the nearest neighbour-matching algorithm and no replacement. The match tolerance was 0.02 in this study. Matching was repeated several times with different random number generator seeds to ensure that the final outcome analysis could produce stable results.

Statistics were generally expressed as the mean values (SD, 95% confidence interval). Proportions and percentages were used to summarize the categorical variables, and medians (range) were used for non-normal variables. Means were compared with independent samples Student’s t-test. The Mann-Whitney U test was used for non-normal data, and categorical variables were analysed with the Pearson χ^2^ test or Fisher exact test. All statistical analyses were performed with SPSS version 23.0 (International Business Machines Corporation, Armonk, NY, USA). Significant differences were defined as when the *p* value was below 0.05.

## Results

**Fig. 3:**
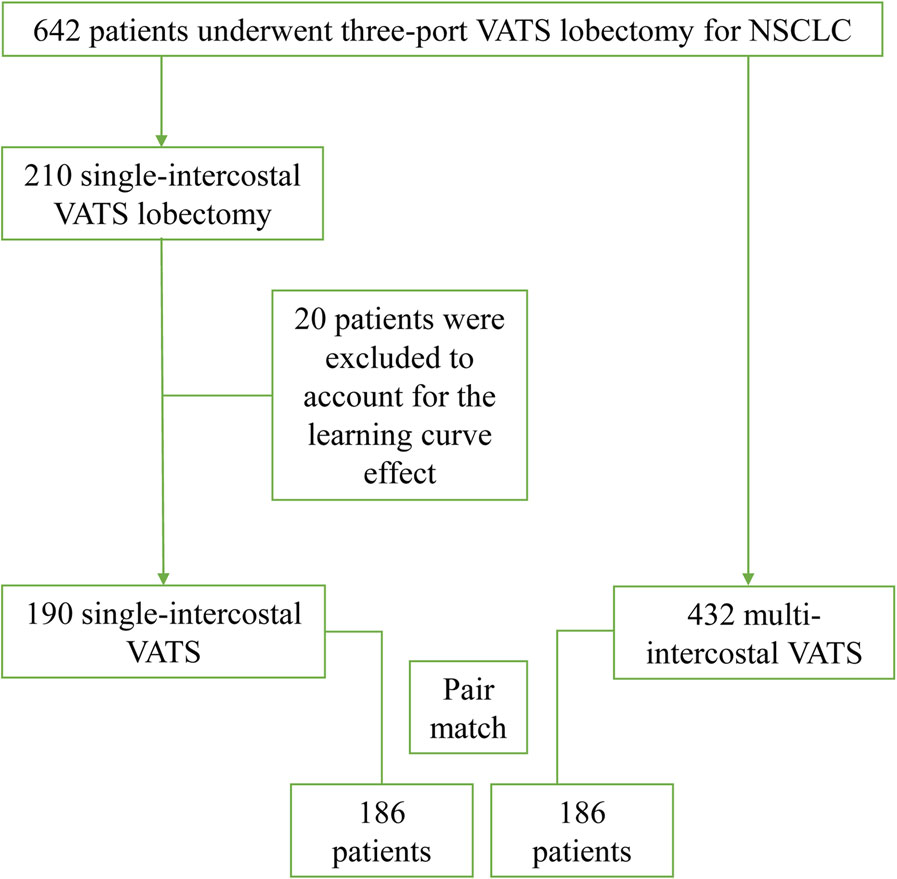
Flowchart summarizing patient enrolment in this study

From January 2013 to January 2018, 642 patients with NSCLC treated with VATS lobectomy were enrolled for analysis in this study (Fig. 3). Among the 642 patients, 210 underwent three-port SIC thoracoscopic lobectomy, and 432 accepted three-port MIC thoracoscopic lobectomy. Patients who were converted to open surgery were included in the final analysis. The first 20 cases in the SIC group were excluded to account for the learning curve effect. A total of 186 closely matched pairs were generated after propensity score matching analysis in this retrospective study. The baseline demographic parameters and clinical characteristics of the study cohort before and after matching are listed in Table 1. Two groups were well matched, and no significant differences were observed in the baseline clinical variables.

**Table 1:** Baseline demographics and characteristics of patients before and after matching

	All patients		*p* value	Propensity-matched Patients		*p* value
	Single intercostal (*n* = 190)	Multi-intercostal (*n* = 432)		Single intercostal (*n* = 186)	Multi-intercostal (*n* = 186)	
Age(years)	59.2 (8.7)	61.3 (9.1)	0.060	59.7 (8.3)	59.9 (8.5)	0.847
Sex			0.221			0.834
Male	106 (55.8%)	218 (50.5%)		104 (55.9%)	106 (57.0%)	
Female	84 (44.2%)	214 (49.5%)		82 (44.1%)	80 (43.0%)	
FEV1 (L)	2.31 (0.43)	2.38 (0.52)	0.193	2.31 (0.43)	2.31 (0.49)	0.986
Smoking history			0.245			0.832
Yes	74 (38.9%)	156 (36.1%)		74 (39.8%)	72 (38.7%)	
No	106 (61.1%)	276 (63.9%)		112 (60.2%)	114 (61.3%)	
BMI(kg/m2)	22.7 (2.8)	23.1 (3.0)	0.313	22.7 (2.8)	23.4 (2.8)	0.112
Tumor size (cm)	2.1 (1.8)	2.4 (1.7)	0.238	2.1 (1.8)	2.2 (1.5)	0.787
Adenocarcinoma	168 (88.4%)	362 (83.8%)	0.135	164 (88.2%)	156 (83.9%)	0.232
Location			0.020			0.321
LUL	32 (16.8%)	70 (16.2%)		28 (15.1%)	28 (15.1%)	
LLL	24 (12.6%)	98 (22.7%)		24 (12.9%)	32 (17.2%)	
RUL	76 (40.0%)	126 (29.2%)		76 (40.9%)	58 (31.2%)	
RML	16 (8.4%)	40 (9.3%)		16 (8.6%)	22 (11.8%)	
RLL	42 (22.1%)	98 (22.7%)		42 (22.6%)	46 (24.7%)	
Pathologic stage			0.050			0.389
I a	140 (73.7%)	278 (64.4%)		136 (73.1%)	133 (71.5%)	
I b	6 (3.2%)	28 (6.5%)		6 (3.2%)	8 (4.3%)	
II a	2 (1.1%)	16 (3.7%)		2 (1.1%)	8 (4.3%)	
II b	20 (10.5%)	46 (10.6%)		20 (10.8%)	14 (7.5%)	
III a	18 (9.5%)	60 (13.9%)		18 (9.7%)	20 (10.8%)	
III b	4 (2.1%)	4 (0.9%)		4 (2.2%)	3 (1.6%)	

Data are presented as n (%) or mean (SD). *FEV1* = forced expiratory volume in 1 second; *BMI* body mass index

A comparison between propensity-matched groups for operative details and perioperative outcomes is shown in Table 2. The mean operating time in the SIC group was 135.6 min (34.0; 95% CI: 130.7–140.9), which was significantly shorter than that of 165.4 min (32.2; 95% CI: 161.0–170.2) in the MIC group (*p* < 0.001). The volume of blood loss was significantly lower in patients in the SIC group than in those in the MIC group (*p* < 0.001). Additionally, the main chest tube (26-Fr) could be removed on postoperative day (POD) 1 from patients in the SIC group, which was significantly faster than in the MIC group (*p* < 0.001). Although the total number of lymph nodes harvested (25.3 vs. 23.8, *p* = 0.160) and stations removed (6.5 vs. 6.7, *p* = 0.368) were similar between the two groups, more subcarinal lymph nodes were removed (6.9 vs. 5.2, *p* < 0.001) in the SIC group than in the MIC group. The length of hospital stay (6.5 vs. 6.3, *p* = 0.478) and the complication rate (4.8% vs. 6.5%, *p* = 0.500) were comparable between the two groups, with no significant differences. Intraoperatively, there were two patients (1.1%) in the SIC group who were converted to thoracotomy due to severe adhesion formation, while four patients (2.2%) in the MIC group were converted to thoracotomy, one for uncontrolled bleeding (injury to the pulmonary artery) and three for severe adhesion in the thoracic cavity. One patient in the MIC group died due to sudden cardiac death on postoperative day 7. No other cases of in-hospital mortality or 30-day mortality were identified in this study.

**Table 2:** Perioperative outcomes of patients in both groups

	Single intercostal (*n* = 186)	Multi-intercostal (*n* = 186)	*p* value
Operative time (min)	135.6 (34.0, 130.7–140.9)	165.4 (32.2, 161.0–170.2)	< 0.001
Blood loss (mL) ^a^	52 (20–350)	100 (40–500)	< 0.001
Conversion	2 (1.1%)	4 (2.2%)	0.681
LN assessment			
Total LN removed	25.3 (11.3, 23.7–26.9)	23.8 (9.7, 22.4–25.1)	0.160
Total LN stations removed	6.5 (1.8, 6.2–6.8)	6.7 (1.9, 6.3–6.9)	0.368
Total subcarinal LN removed	6.9 (3.8, 6.4–7.5)	5.2 (3.5, 4.7–5.7)	< 0.001
Chest tube removal (26-Fr) (PODs)	1 (0–18)	3 (2–12)	< 0.001
Length of stay (days)	6.5 (3.1, 6.1–7.0)	6.3 (3.3, 5.8–6.8)	0.478
Major complications			
Chylothorax	2 (1.1%)	1 (0.5%)	1.000
Atelectasis	3 (1.6%)	7 (3.2%)	0.200
Pulmonary embolism	1 (0.5%)	0 (0.0%)	1.000
Empyema	1 (0.5%)	2 (1.1%)	1.000
Reoperation	2 (1.1%)	2 (1.1%)	1.000
Total	9 (4.8%)	12 (6.5%)	0.500
Mortally	0 (0.0%)	1 (0.5%)	1.000

Data are presented as median (range) or mean (SD, 95% confidence interval). *LN* lymph nodes; *PODs* postoperative days; a: skewed distribution, Mann-Whitney U test applied

We also analysed whether the perioperative outcomes (operative time, blood loss and length of stay) in the three-port SIC group varied with the preoperative variables (age, sex, tumour location, number of lymph nodes harvested and tumour size) and found that only patients who were older than 30 had a longer hospital stay (*p* = 0.039) (Table 3).

**Table 3:** Comparison of clinical variables and perioperative outcomes of single-intercostal thoracoscopic lobectomy (n = 186)

Variable	n	Operative time (min)	Blood loss (mL) ^a^	Length of stay (day)
Age (years)				
< 60	88 (47.3%)	128.3 (28.2)	50 (20–205)	5.8 (2.8)
≥ 60	98 (52.7%)	142.2 (37.8)	58 (20–350)	7.2 (3.2)
		*p* = 0.056	*p* = 0.153	*p* = 0.039
Sex				
Male	104 (55.9%)	137.4 (35.1)	54 (20–200)	7.0 (3.5)
Female	82 (44.1%)	133.4 (33.1)	50 (20–350)	6.0 (2.4)
		*p* = 0.576	*p* = 0.819	*p* = 0.121
Anatomic resection				
Upper lobe	104 (55.9%)	140.4 (38.4)	51 (20–350)	6.1 (2.2)
Lower/middle lobe	82 (44.1%)	129.6 (27.1)	53 (20–200)	7.1 (3.9)
		*p* = 0.118	*p* = 0.792	*p* = 0.187
Extensive lymphadenectomy ^b^				
Yes	116 (68.4%)	131.6 (33.8)	51 (20–200)	6.8 (3.6)
No	70 (37.6%)	142.3 (34.1)	55 (20–350)	6.1 (1.9)
		*p* = 0.145	*p* = 0.408	*p* = 0.241
Tumor size (cm)				
< 3	142 (76.3%)	134.6 (35.0)	50 (20–350)	6.5 (3.3)
≥ 3	44 (23.7%)	139.1 (31.8)	58 (49–200)	6.8 (2.3)
		*p* = 0.594	*p* = 0.244	*p* = 0.630

Data are presented as median (range) or mean (SD). a: Skewed distribution, Mann-Whitney U test applied; b: more than 20 lymph nodes harvested in patients

## Discussion

In this retrospective study, we demonstrated that compared with NSCLC patients treated with three-port MIC lobectomy, those treated with three-port SIC lobectomy had a potential advantage with regard to less direct stimulation of the intercostal nerves and more subcarinal lymph nodes removed. In addition, there was no compromise in the total number of lymph nodes harvested, complications or length of hospital stay.

Numerous studies have demonstrated that conventional three-port MIC VATS lobectomy is an acceptable procedure for the treatment of NSCLC with a low morbidity and mortality rate [2–4]. The Society of Thoracic Surgeons database reported (*n* = 1281) that for VATS lobectomy, the total complication rate was 26.2%, and the mortality rate was 0.94% [3]. A case-control study by McKenna et al. [12] presented (*n* = 1100) that the total complication rate was 15.3%, and the mortality rate was 0.8%. In previous study [10], we reported our initial results of three-port SIC VATS for bulla resection, wedge resection, segmentectomy and lobectomy. However, three-port SIC and MIC VATS lobectomy have never been compared before. In this study, we found that a small percentage of patients had major morbidity: 4.8% of patients in the SIC group and 6.5% of patients in the MIC group. There was no significant difference in major morbidity between the two groups. There was only one death (0.5%) in the MIC group, and no mortality was recorded in the SIC group during this hospitalization. Accordingly, we can conclude that three-port SIC and MIC lobectomy were both safe procedures that could be performed by experienced thoracic surgeons and conferred low morbidity among patients with NSCLC.

The potential advantage of SIC lobectomy is that only one intercostal space was used to complete the operation, which could theoretically reduce the damage to the intercostal nerves and result in less pain. However, we did not compare pain scores between the two groups in this study since we have continually made effort to reduce postoperative pain for patients in recent years. According to previous studies [13, 14], acute pain following VATS was mainly due to intercostal nerve damage, surgical trauma to muscles and placement of the chest tube. In addition, the ideal anaesthetic regimen for VATS would be to use regional anaesthesia combined with opioids and nonopioid analgesics [13]. Thus, reducing direct stimulation of the intercostal nerves, removing the main chest tube earlier and using patient-controlled paravertebral blocks for continued regional anaesthesia are key strategies for reducing postoperative pain. We speculate that SIC lobectomy may result in less postoperative pain and better patient tolerance than MIC lobectomy.

Radical systemic mediastinal lymphadenectomy (more than 20) was routinely performed for most patients with NSCLC in our department. In this study, the total number of lymph nodes harvested (25.3 vs. 23.8) and stations removed (6.5 vs. 6.7) were similar between the two groups. It has been reported that radical mediastinal lymphadenectomy can provide accurate pathologic staging and is probably advantageous for survival outcomes [15]. The disease-free and overall survival of patients can improve, with a plateau at 11 or more lymph nodes [16, 17]. Wang et al. [5] and Shen et al. [18] reported that approximately 22 lymph nodes were harvested for conventional three-port MIC VATS, which was similar to the number of lymph nodes harvested in MIC VATS in this study.

Furthermore, more subcarinal lymph nodes were removed (*p* < 0.001) in the SIC group than in the MIC group. Lymphadenectomy has become easier for surgeons to perform over time. Multiple ports provide different angles for lymphadenectomy. Moreover, the surgeon can directly visualize the target tissue (subcarinal lymph nodes) when performing three-port SIC lobectomy because the camera port in the posterior axillary line at the sixth or seventh intercostal space can provide more direct and accurate visualization of the target structure in SIC VATS (Fig. 2) than can the observation port created in the lower intercostal space in the conventional three-port MIC VATS. Therefore, more subcarinal lymph nodes were harvested and more radical systemic mediastinal lymphadenectomies were performed in patients treated with the modified VATS technique.

There were two factors contributing to the shorter surgery duration and the reduced intraoperative blood loss for SIC VATS compared with conventional three-port MIC VATS. One factor was that positioning patients in the lateral decubitus position allows the surgeon to have direct visualization and to perform direct instrumentation and dissection from the sixth intercostal space, which is very similar to thoracotomy. A major factor was that no extra instrument was needed, and an operating angle between the instruments still existed. Thus, there are no obstacles in converting from the MIC to the SIC approach for thoracic surgeons with experience in minimally invasive surgery. Additionally, 20 cases were initially excluded to account for the learning curve effect in this study. With increasing experience, the operative time and blood loss associated with three-port SIC lobectomy decreased.

Our approach for SIC VATS lobectomy in this study has the following characteristics: (a) a single intercostal space is used to complete the operation; (b) accurate anatomic visualization is achieved for subcarinal lymph nodes removed from the posterior axillary line at the sixth intercostal space, which is highly similar to thoracotomy; (c) no extra instrument is needed, and the operating angle between the instruments still exists; and (d) it is feasible for thoracic surgeons with experience in minimally invasive surgery to convert from the MIC approach to the SIC approach.

There are several limitations to this study. First, the major limitation is the retrospective design and the decision to perform MIC or SIC is based on surgeon preference. Although propensity-matched analysis may reduce some selection bias, inherent selection bias and unknown confounding variables cannot be eliminated. Second, the experience in this study was from two experienced surgeons in a high-volume thoracic department. Third, the theoretical benefit of SIC versus MIC with regard to pain management could not be investigated in this study, as many changes in postoperative management (analgesics, chest tube and intercostal nerve damage) complicated controlling for biases, even with propensity matching. Thus, a prospective and randomized multicentre study would be the gold standard to demonstrate the superiority of one procedure over another, and such a study will be conducted in the near future.

## Conclusions

Based on the presented data, we conclude that three-port SIC lobectomy and MIC lobectomy are both acceptable and offer similar oncologic surgery for the treatment of NSCLC. Thus, three-port SIC provides another choice for the treatment of NSCLC, and surgeons can freely choose with strong self-confidence according to preference.

## Abbreviations

CI: confidence interval
FEV1: forced expiratory volume in the first second
MIC: multiple-intercostal
NSCLC: non-small-cell lung cancer
POD: postoperative day
SIC: single-intercostal
VATS: video-assisted thoracoscopic surgery
